# Modulation of inhibitory control networks relate to clinical response following ketamine therapy in major depression

**DOI:** 10.1038/s41398-020-00947-7

**Published:** 2020-07-30

**Authors:** Ashish K. Sahib, Joana RA. Loureiro, Megha M. Vasavada, Antoni Kubicki, Benjamin Wade, Shantanu H. Joshi, Roger P. Woods, Eliza Congdon, Randall Espinoza, Katherine L. Narr

**Affiliations:** 1 Department of Neurology, Ahmanson-Lovelace Brain Mapping Center, Los Angeles, CA USA; 2 Department of Psychiatry and Biobehavioral Sciences, University of California Los Angeles, Los Angeles, CA USA

**Keywords:** Diagnostic markers, Predictive markers

## Abstract

Subanesthetic ketamine is found to induce fast-acting and pronounced antidepressant effects, even in treatment resistant depression (TRD). However, it remains unclear how ketamine modulates neural function at the brain systems-level to regulate emotion and behavior. Here, we examined treatment-related changes in the inhibitory control network after single and repeated ketamine therapy in TRD. Forty-seven TRD patients (mean age = 38, 19 women) and 32 healthy controls (mean age = 35, 18 women) performed a functional magnetic resonance imaging (fMRI) response inhibition task at baseline, and 37 patients completed the fMRI task and symptom scales again 24 h after receiving both one and four 0.5 mg/kg intravenous ketamine infusions. Analyses of fMRI data addressed effects of diagnosis, time, and differences between treatment remitters and non-remitters. Significant decreases in brain activation were observed in the inhibitory control network, including in prefrontal and parietal regions, and visual cortex following serial ketamine treatment, *p* < 0.05 corrected. Remitters were distinguished from non-remitters by having lower functional activation in the supplementary motor area (SMA) prior to treatment, which normalized towards controls following serial ketamine treatment. Results suggest that ketamine treatment leads to neurofunctional plasticity in executive control networks including the SMA during a response-inhibitory task. SMA changes relate to reductions in depressive symptoms, suggesting modulation of this network play an important role in therapeutic response. In addition, early changes in the SMA network during response inhibition appear predictive of overall treatment outcome, and may serve as a biomarker of treatment response.

## Introduction

Major depression is the world’s leading cause of years lost to disability^1^. Although many patients benefit from first-line monoaminergic antidepressants, therapeutic response can take weeks or longer^2^ and a third of patients, defined as having treatment resistant depression (TRD), will remain refractory to two or more treatment trials^3,4^. Therefore, understanding of rapid response mechanisms remains pivotal for advancing more effective interventions to reduce the personal and economic burden of depression. Ketamine is a noncompetitive N-methyl-D-aspartate (NMDA) receptor antagonist, which when administered at sub-anesthetic doses, is shown to produce fast and robust antidepressant effects in patients with TRD^5^. However, though ketamine, delivered in either its racemic form of *S*(+) and *R*(−) enantiomers^6,7^, or as *(S)-*ketamine only^8,9^, can reduce depressive symptoms within hours, relatively little is known of its effects on brain function at the systems-level following single or repeated doses. Indeed, only a handful of published neuroimaging studies have addressed how low-dose ketamine influences dimensions of function potentially underlying symptom improvement in TRD^10–14^. Further, these studies have either explored resting-state function^10,11^, or employed brain activation tasks to examine aspects of emotion processing specifically^12–14^. The influence of ketamine on cognitive systems, which regulate emotion and other cognitive functions, remain mostly unknown.

Disturbances in cognitive processes that oversee the regulation of environmental cues or thoughts to adjust or inhibit behavior are considered central to the pathophysiology of major depression^15,16^. Accumulating evidence also supports that disturbances of cognitive control, which are widely observed in depression^17,18^, are linked with the top–down dysregulation of prefrontal and parietal neural circuitry^19,20^. Further, neurofunctional changes in the cognitive control network^21^ are shown to relate to or be predictive of clinical outcomes following treatment with slower-acting monoaminergic pharmacotherapies^22–24^. Both interference and response inhibition tasks are frequently used to probe inhibitory control circuitry, where response inhibition is considered a subconstruct of cognitive control according to the Research Domain Criteria Matrix^25^. For response inhibition or inhibitory control in particular, Go/NoGo tasks, which require withholding responses for certain items in a series of stimuli^26–29^, elicit reproduceable activations in cognitive-control-related frontal and parietal regions, connected association regions such as the supplementary motor (SMA) cortex, and the striatum, thalamus, and cerebellum^21,30–33^. As may be dependent on task demands, analysis methods, and patient characteristics, such as comorbidity for anxiety disorders, both increased activity in frontal regions^22,34^, as well as hypo-frontal activation during inhibitory processes have been reported in MDD^15,35^. Cognitive regulation abilities for both emotionally neutral or valenced stimuli may thus be important for recovery of depressive symptoms^36–38^.

To determine whether ketamine modulates dysfunctional inhibitory mechanisms reported in major depression^17,18^, the present study used an event-related design during a response inhibition Go/NoGo functional magnetic resonance imaging (fMRI) task in TRD patients followed prospectively through a series of four subanesthetic intravenous (IV) ketamine treatments. We examined whether NoGo > Go blood-oxygen-level dependent (BOLD) activity associated with remission differs between MDD and HC, and whether it changes with ketamine treatment. Based on previous findings^23,24^, we hypothesized that response-inhibitory activity would likely dissociate remitters from remitters, and that regional brain activity would differ following ketamine treatment.

## Methods and materials

### Subjects

**Fig. 1:**
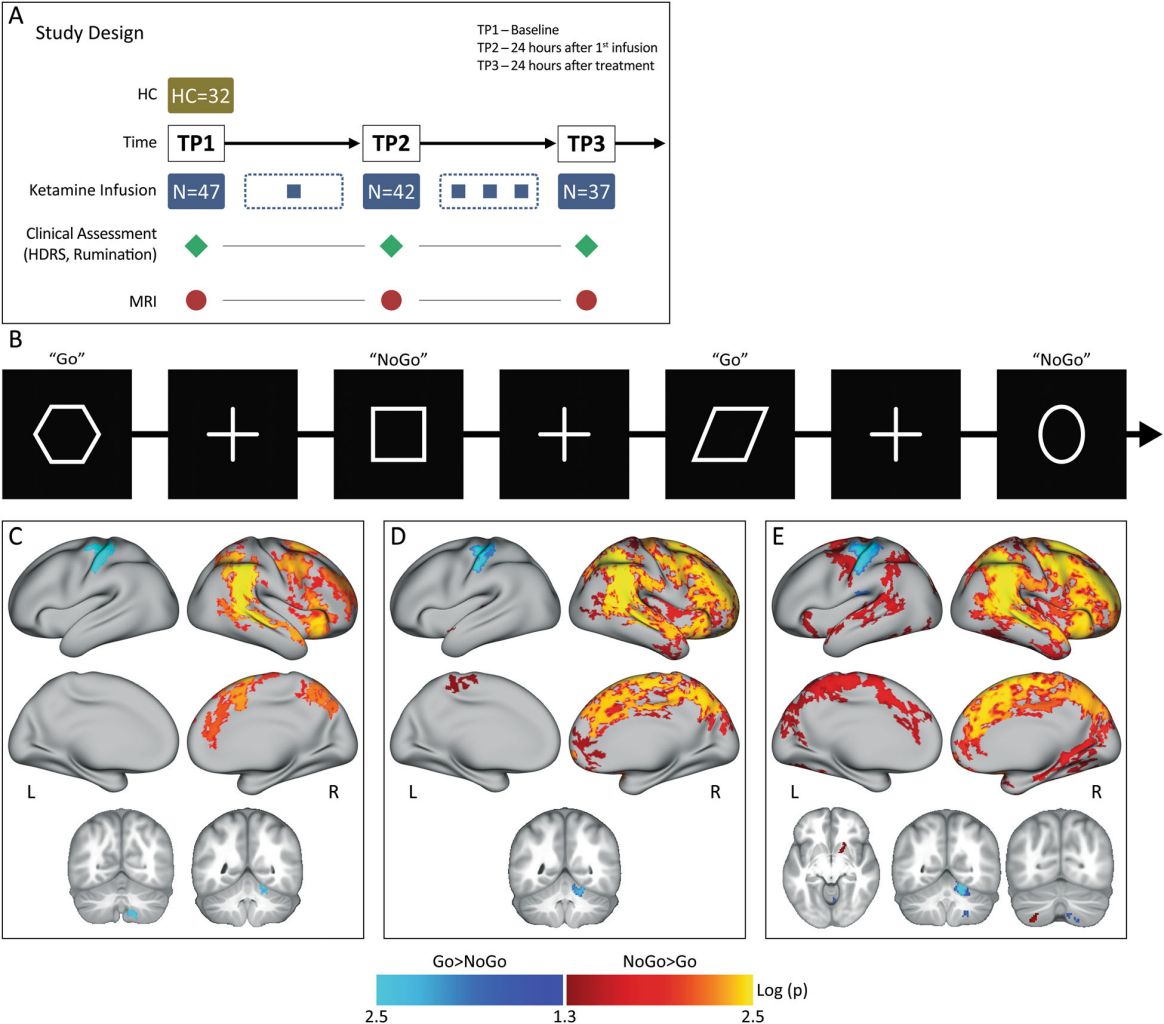
Experimental design and task. **a** Study design illustrating the timing of MRI sessions and clinical assessments relative to ketamine infusions. **b** Inhibitory Control task. Participants view a series of shapes, and are instructed to press a button for all shapes presented (i.e., Go stimuli) with the exception of circles and squares (i.e., NoGo stimuli). **c** Group activation maps of the response inhibition contrast in HC, **d**, TRD, **e** and both HC and TRD participants. Positive activations (hot colors) depict NoGo>Go activity, negative activations (cool colors) depict Go>NoGo activity. Images are thresholded at log(p) >1.3, which corresponds to *p* < 0.05, FWE (TFCE) corrected across grayordinates. Coronal and axial images are in neurological convention (R = R). Results are displayed in CIFTI surface space. L = left, R = right.

Participants included 32 healthy controls (HC) and 47 DSM–5 defined (SCID^39^,) individuals who met criteria for TRD (i.e., failed ≥2 adequate antidepressant trials and had been continuously depressed for ≥6 months, all 20–64 years of age). Subjects were recruited from the Los Angeles area through advertisements, clinician referral or clinicaltrials.gov (NCT02165449). TRD subjects were followed prospectively during a series of four ketamine treatments. Imaging and clinical assessments occurred at three timepoints: (1) initial baseline occurring within 1 week of the first treatment (TP1, *N* = 47); (2) 24 h after the first ketamine infusion (TP2, *N* = 37) and; 24–72 h after the final ketamine infusion (TP3, *N* = 37) (Fig. 1a). At each time point, depression severity was assessed using the Hamilton Depression Rating Scale, 17–item (HDRS)^39,40^, which was used as the primary measure of antidepressant response. Patients whose HDRS score reached ≤ 7 at the end of treatment (TP3) were considered as remitters, with the remainder of patients defined as non-remitters. Demographic and clinical information is provided in Table 1.

**Table 1:** Patient demographic and clinical information.

	HC mean (SD)	MDD mean (SD)	*T*/*χ*	*p*
Number of subjects (*N*)	32	47	N/A	N/A
Gender (% female)	56.25	40.42	$\chi$ = 1.91	0.17
Age (years)	34.75 (13.5)	38.61 (10.6)	*T* = 0.52	0.6
Education (years)	10.42 (2.0)	10.15 (2.3)	*T* = −1.4	0.16
Duration lifetime illness (years)	N/A	21.65 (12.32)	N/A	N/A
Current episode (years)	N/A	5.3 (6.51)	N/A	N/A
^a^Comorbid disorders				
Generalized anxiety	N/A	31	N/A	N/A
Manic episodes	N/A	0	N/A	N/A
Feeding and eating disorders	N/A	4	N/A	N/A
Trauma and stressor related disorders	N/A	13	N/A	N/A
ADHD	N/A	1	N/A	N/A
Somatic Symptom and related disorders	N/A	1	N/A	N/A
HDRS (TP1)	N/A	20.06 (4.1)	N/A	N/A
HDRS (TP2)	N/A	13.47 (4.3)	N/A	N/A
HDRS (TP3)	N/A	8.91 (4.5)	N/A	N/A

*HDRS* Hamilton Depression Rating Scale (HDRS), *HC* healthy controls, *TP1* patients with major depressive disorder (MDD) at baseline, *TP2* 24 h after the first infusion and *TP3* after fourth infusion that included 37 participants.

^a^Comorbid disorders based on SCID -V.

Exclusion criteria for all participants included any unstable medical or neurological condition, current substance abuse or dependence (ascertained by laboratory testing) or substance abuse history within the preceding 3-months, current or past history of psychosis, schizophrenia, mental retardation or other developmental disorder, diagnosis of dementia and any contraindication to scanning (e.g., metal implants or claustrophobia). At baseline, patients had moderate to severe depressive symptoms as per the HDRS^40^ (baseline HDRS ≥ 17). Subjects were also screened to ensure no prior psychotic reactions to medications, alcohol or illicit substances in the past, and for other physical or clinical contraindications to ketamine. All subjects provided written informed consent following procedures approved by the University of California, Los Angeles (UCLA) Institutional Review Board.

### Ketamine treatment

Patients receiving ketamine treatment were permitted to remain on approved monoaminergic antidepressant therapy (if unchanged in the preceding 6-weeks) for the duration of the study (Supplementary Table S1). Benzodiazepines were held the night before and morning of all study visits (e.g., scan sessions, ketamine infusion session). Patients received infusions 2–3 times a week for a total of four infusions. At each session, performed as an outpatient procedure, a single sub-anesthetic dose (0.5 mg/kg) of ketamine diluted in 60 cc normal saline was delivered intravenously via pump over a 40-min period in a private room at the UCLA Clinical Translational Research Center or the Resnick Neuropsychiatric Hospital. Vital sign monitoring included blood pressure, pulse oximetry, and respiratory rate recording every 3 min and a continuous cardiac rhythm strip. Mental status monitoring also occurred during ketamine infusion to assess for any untoward behavioral or psychological effects.

### Functional imaging task

Participants performed a Go/NoGo task that probes inhibitory control processes. This “CARIT” task (Conditioned Approach Response Inhibition Task) is identical to the Go/NoGo task used in Human Connectome Project (HCP) Lifespan studies for Aging and Development^41,42^. Participants are instructed to rapidly press a button in response to seeing a shape (“Go”) but withhold responses for squares and circles (“NoGo”). Button presses are attributed to a given trial if they occur within 800 ms of stimulus onset, which includes 600 ms stimulus duration and 200 ms fixation (Fig. 1b). Response data records accuracy and reaction time for both GO and NoGo trials.

### MRI data acquisition

A Siemens 3 T Prisma MRI system at UCLA’s Ahmanson-Lovelace Brain Mapping Center was used to acquire imaging data with a 32-channel phased array head coil. Acquisition sequences were identical to those used by the HCP Lifespan studies for Aging and Development (https://www.humanconnectome.org)^43^. Structural scans consisted of a T1-weighed (T1w) multi-echo MPRAGE (voxel size (VS) = 0.8 mm isotropic; repetition time (TR) = 2500 ms; echo time (TE) = 1.81:1.79:7.18 ms; inversion time (TI) = 1000 ms; flip angle (34) = 8.0°; acquisition time (TA) = 8:22 min) and a T2-weighted (T2w) acquisition (VS = 0.8 mm isotropic; TR = 3200 ms; TE = 564 ms; TA = 6:35 min), both with real-time motion correction^44^. Functional imaging data was acquired using a multiband EPI sequence with voxel size (VS) = 2 mm isotropic; repetition time (TR) = 800 ms; echo time (TE) = 37 ms, flip angle (FA) = 52°, MB accl. factor = 8; phase enc. direction = PA; total acquisition time (TA) = 4.00 min.

### MRI data analysis

Imaging data was preprocessed using the HCP minimal pipelines^45^, which included registration into CIFTI space (combined cortical surface and subcortical volume coordinate system) implemented within the BIDS-App^46^. Following preprocessing, functional images were denoised using FSL FIX (https://fsl.fmrib.ox.ac.uk/fsl/fslwiki/FIX). Smoothing (5 mm) was applied to the preprocessed images using the grayordinates-based approach^47^. The quality of the functional images was assessed by visually inspecting the ICA components for each subject separately. Images with artifacts remaining after FIX, which comprised of one subject not counted in the reported sample size, were excluded from the study. Following preprocessing, a general linear model (GLM) estimated task effects at the whole-brain level with four task regressors (correct Go, correct NoGo, incorrect NoGo and incorrect Go). Since the NoGo>Go contrast identifies brain regions involved in response inhibition, within and across group analyses targeted this statistical contrast for correct trials. To characterize patterns of neural response based on inhibitory control demands, one-sample t-tests were used to generate average activation maps within HC and TRD groups, and in both groups combined (HC+TRD) at TP1, including age and sex as covariates.

Subsequent group-level analyses included examination of (1) cross-sectional effects between diagnostic groups at baseline, (2) longitudinal effects of ketamine treatment, and (3) differences between patients defined as treatment remitters and non-remitters. Post-hoc analyses addressed correlations between change in neural response occurring after single or serial ketamine treatment and antidepressant response. First, a two-sample t-test with age and sex as regressors of no interest compared cross-sectional differences in whole-brain activation between HC and TRD at baseline. Second, to test for longitudinal effects of ketamine treatment, paired t-tests compared whole-brain contrast maps between time points examined pairwise, evaluating effects of both single (TP1–TP2) and serial ketamine treatment (TP1-TP3). Third, to test whether NoGo>Go fMRI activity relates to treatment outcome, two-sample t-tests (with age and sex as covariates) were used to compare change in activity between patients defined as remitters and non-remitters using whole-brain ΔNoGo > Go contrast maps (TP1 - TP3). For all whole-brain analyses, nonparametric permutation testing (5000 permutations) were implemented with FSL’s PALM^48^ to correct for multiple comparisons. Statistical thresholds were set at threshold-free cluster estimates (TFCE) with family-wise error corrected (FWE) at *p* < 0.05^49^. Mean contrast (NoGo vs Go) values from ROIs were used to visualize the direction of change in activation (qualitative) for whole brain analyses, and for follow-up analyses described below. Here, ROIs were masked based on the intersection of clusters that survived statistical significance (TFCE, FWE *p* < 0.05) for tests of remitter status with anatomical labels derived from the Freesurfer (Desikan–Killiany) atlas^50^.

For follow-up analyses, mean ROI contrast values were used as dependent measures to confirm the presence of time-by-remission status interactions employing a general linear mixed model with time (TP1 and TP3) and remitter status (remitter, non-remitter) as fixed factors. Post-hoc analyses were also performed to map relationships between changes in BOLD response and percent change in HDRS. Finally, mean ROI contrast values were used to establish whether neural response after single infusion (TP1–TP2) relates to percent change in HDRS scores after serial ketamine (TP1–TP3). To investigate the change in response inhibitory activity post ketamine infusion across large scale networks, we also extracted BOLD activity for the NoGo>Go contrast from four large scale networks using the Yeo 7-network atlas^51^ (Supplementary Fig. S1). All ROI-level analyses were performed using the IBM Statistical Packages for the Social Sciences (SPSS v25) with a *p* value of <0.05 as the threshold statistical significance.

## Results

### Demographic and clinical results

Age and sex did not significantly differ between HC and TRD groups at baseline (Table 1). HDRS (*F* (2, 65.70) = 77.48, *p* < 0.0001) scores showed significant improvement across time (Table 1) and maximum improvement occurred after serial ketamine infusion (TP3). Of the 37 TRD patients that completed four ketamine infusions, 15 (40%) achieved remission (HDRS < 7).

### Response variables

There were no significant differences across time for response variables including total, Go or NoGo trial reaction time or accuracy in TRD patients (all *p* > 0.05). Differences between HC and patients at baseline (TP1) for task response variables were also absent (all *p* > 0.05).

### Within group activation of the inhibitory control network

The average activation maps for the NoGo>Go contrast within HC, TRD, and combined HC+TRD groups at baseline are shown in Fig. 1c–e. These activation maps illustrate response inhibition associates with a predominantly right-lateralized functional network (TFCE, FWE *p* < 0.05). Specifically, significantly greater activity was observed in the inferior frontal and dorsolateral prefrontal cortex (DLPFC), and the inferior and superior parietal regions. For the Go>NoGo contrast, which was not the focus of this study, we observed significantly greater activity in the left motor cortex and right cerebellum (participants used their right hand to make button presses). For the combined sample of TRD and HC participants (*N* = 79), we also observed increased NoGo>Go activity in the right putamen and left cerebellum Fig. 1e.

### Cross-sectional effects between diagnostic groups at baseline

Despite the greater activations observed in the NoGo>Go response inhibition network in TRD patients relative to controls at baseline (Fig. 1d), these effects did not survive *p* < 0.05 FWE correction at the whole-brain level. Nonetheless, regional values from HC were used as a guide to establish whether effects associated with ketamine treatment approached normalization.

### Longitudinal effects of ketamine treatment

**Fig. 2:**
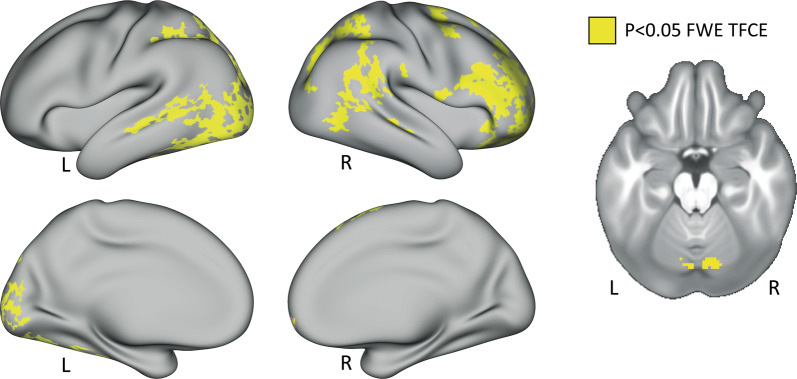
Effects of serial ketamine. Effect of ketamine treatment after four serial infusions (TP1vsTP3). Clusters in yellow correspond to *p* < 0.05, FWE (TFCE) corrected across grayordinates. Results are displayed in CIFTI surface space. L = left, R = right.

The paired *t*-test comparing the NoGo>Go contrast at baseline (TP1) and after four ketamine infusions (TP3) showed significant network-related decreases in activation over time (Fig. 2). Regions involved in the inhibitory response network, including the inferior frontal cortex and DLPFC along with the superior and inferior parietal regions and the right cerebellum, showed a significant decrease in activation at TP3 compared to TP1. In addition, the visual cortex and superior parietal regions of the left hemisphere showed a significant decrease in activation at TP3. The paired *t*-tests addressing effects of single ketamine (TP1–TP2), and comparing TP2 and TP3 did not reveal any significant differences after *p* < 0.05 FWE (TFCE) correction. Effects of the Go>NoGo contrast were also examined in follow-up analysis, but no significant differences were observed between ketamine treatment time points. In terms of large-scale networks, we observed a significant decrease in BOLD activity for the default mode network (DMN), fronto-parietal network (FPN), dorsal-attention network (DAN), and the salience network (SAN) in the right hemisphere following ketamine treatment (Supplementary Fig. S1).

### Effects of clinical outcome

**Fig. 3:**
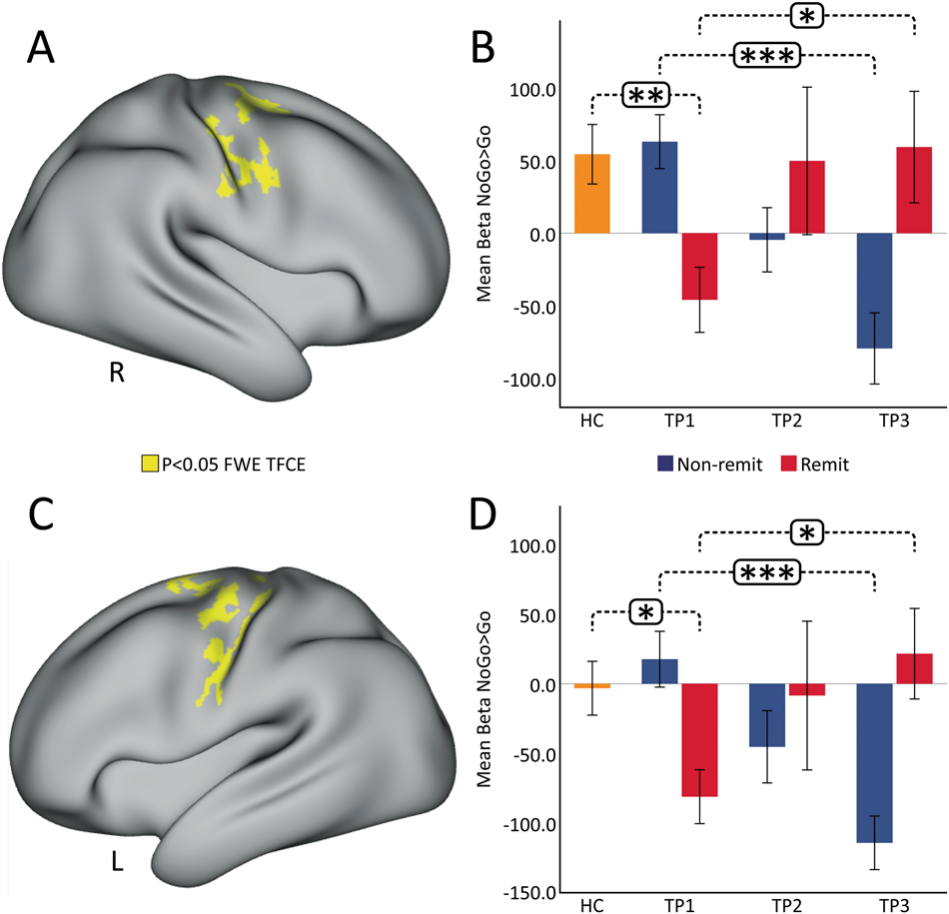
Effects of Remitter status. Whole brain activation maps showing results from two-sample t-test for change in BOLD (TP1–TP3) activity between remitters and non-remitters. Clusters in yellow correspond to *p* < 0.05, FWE (TFCE) corrected across grayordinates for the right **a** and left **c** SMA. Results are displayed in CIFTI surface space. The bar plots represent the average Betas for the NoGo > Go contrast in the right **b** and left **d** SMA across ketamine treatment (*p < 0.05, **p < 0.01, ***p < 0.001). R = right. L = left, R = right.

Significant changes were observed in ΔNoGo > Go contrast maps between remitters and non-remitters post serial ketamine treatment (TP1–TP3) across the somato-motor network (Fig. 3a, c). In particular, the change was significant (*p* < 0.05 FWE) in the right and left precentral gyrus. Furthermore, there was significant time-by-remission status interaction of BOLD activity in the right (*F*(2) = 10.963, *p* < 0.001) and left (*F*(2)=10.905, *p* < 0.001) precentral gyrus. Bar plots show that the mean NoGo>Go activity in the right (Fig. 3b) as well as the left (Fig. 3d) precentral gyrus for non-remitters (blue bars) is similar to HC (orange bars) at baseline (TP1), which significantly decrease with ketamine treatment. Remitters on the other hand (red bars in Fig. 3b, c) showed lower NoGo>Go activity as compared to HC at baseline that significantly increased with ketamine treatment.

### Post-hoc ROI analysis of associations with HDRS

**Fig. 4:**
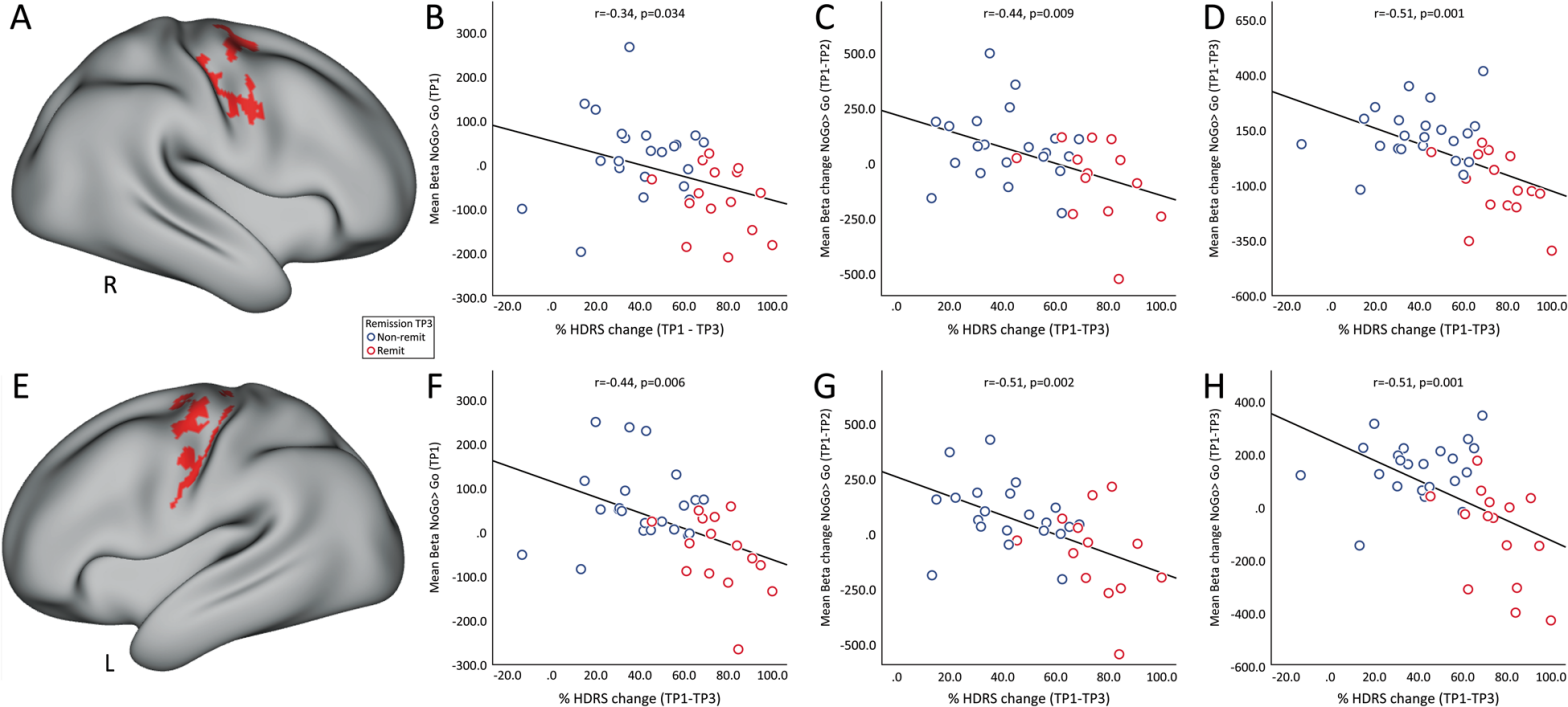
Associations with clinical outcome. Average regional NoGo>Go contrast values in the right (**a**) and left (**e**) SMA ROI (in red) showed a significant association with HDRS scores. **b** Baseline (TP1) average regional NoGo>Go contrast values in the right SMA showed a significant negative relationship with serial change in HDRS (TP1–TP3); **c** Acute change (TP1-TP2) of average regional NoGo>Go contrast values in the right SMA showed a significant negative relationship with serial change (TP1–TP3) HDRS scores. **d** Serial change (TP1-TP3) of average regional NoGo>Go contrast values in the right SMA showed a significant negative relationship with serial change (TP1–TP3) HDRS scores. Similarly, **f** Baseline (TP1) average regional NoGo>Go contrast values in the left SMA showed a significant negative relationship with serial change in HDRS (TP1–TP3); **g** Acute change (TP1–TP2) of average regional NoGo>Go contrast values in the left SMA showed a significant negative relationship with serial change (TP1–TP3) HDRS scores. H) Serial change (TP1–TP3) of average regional NoGo>Go contrast values in the left SMA showed a significant negative relationship with serial change (TP1–TP3) HDRS scores R = right. L = left.

Figure 4a, e show the ROIs selected that map the right and left precentral gyrus. The mean NoGo>Go contrast values from these ROIs was used to determine associations with HDRS scores. Average contrast values in both the ROIs at baseline (TP1) showed significant negative correlation with % change (TP–TP3) in HDRS scores after serial treatment (Fig. 4b, f). These correlations were also observed between acute change (TP–TP2) of mean NoGo>Go contrast values in the selected ROI with serial % change (TP–TP3) in HDRS scores (Fig. 4c, g). In addition, decrease in contrast values in the right and left precentral gyrus 24 h post serial ketamine infusion showed a significant correlation with serial (TP1–TP3) improvement in HDRS scores (Fig. 4d, h).

## Discussion

The relatively recent discovery^52^ and replication of ketamine’s antidepressant properties^53–58^ have provided a major breakthrough towards advancing more effective interventions for TRD. However, both the molecular mechanisms, which appear to extend beyond NMDA receptor antagonism, and the brain systems-level mechanisms accounting for changes in behavior linked with therapeutic response remain unclear^59,60^. The current investigation thus sought to address how ketamine modulates inhibitory control networks in the brain in relation to antidepressant response. Specifically, using non-invasive fMRI and well-validated response-inhibition task (the CARIT task^42^), the present study examined how single and repeated subanesthetic administration of ketamine modulates inhibitory control networks in TRD over time. Our primary results revealed that serial ketamine infusion induces a robust decrease in fMRI activity in brain regions associated with response inhibition^21,33^, including DLPFC along with areas in the superior and inferior parietal lobules. Furthermore, this significant decrease in fMRI activity was observed across various large-scale networks^51^ such as the DMN, FPN, DAN, and the SAN in the right hemisphere following ketamine treatment (Supplementary Fig. S1). We did not observe any significant differences in the response variables between HC and TRD, which may be due to the simple design of the CARIT task. Further, results showed that the patterns of decreased activation in the bilateral precentral gyrus after serial ketamine infusion occur in relation to remission of depressive symptoms and change in the direction of normative values. We also show that early change in the SMA predicts the extent of response following repeated treatments. Overall, these findings suggest that repeated ketamine infusion normalizes neural dysfunction underlying motor inhibitory control in TRD patients, and this normalization relates to symptom improvement.

As expected from prior studies on the inhibitory control network in healthy^32^ and clinical populations^15^, we observed more pronounced right-hemisphere activation of brain regions in this functional circuit. A core set of commonly activated regions among HC and TRD participants included the DLPFC, and the dorsal anterior cingulate cortex, though qualitatively activations appeared greater and more widespread in patients. In addition, increased inhibitory control-related activity was observed in parietal, insula and SMA regions. The inferior parietal cortex has extensive reciprocal connections with the prefrontal cortex^61,62^ and these connections are crucial for executive control needed to guide stimulus-driven attention^63^. Activation of the insula, also present in both HC and TRD groups, may relate to working memory and selective visual attention task demands^64^. The observed activation of the SMA is consistent with electrophysiological, human lesion and functional neuroimaging studies that support the crucial role of SMA in response inhibition^65–67^.

Although effects were not significant across diagnostic groups at the whole-brain voxel-level in cross-sectional comparisons, more powerful longitudinal analyses in TRD demonstrate that significant change of BOLD activity occur in brain regions responsible for inhibitory control 24 h after receiving serial ketamine treatment. In particular, we observed a significant time-by-remission interaction of BOLD activity in the bilateral precentral gyrus. Remitters showed lower BOLD activity in the SMA as compared to HC at baseline that increased with ketamine treatment, while, non-remitters showed increased or similar BOLD activity to HC at baseline that decreased with ketamine treatment. MDD patients are known to show disrupted functional integration in the SMA, which may be due to psychomotor retardation and has been characterized as a key feature of MDD^68,69^. Growing evidence also suggests that SMA plays a vital role in human executive functions, and integration of affective, behavioral and cognitive functions^70^. This disrupted functional activation in the SMA is shown to be modulated with standard antidepressants^71^, and TRD patients with hypometabolism in the SMA are known to favorably respond to antidepressant treatment^72^. Furthermore, a single dose of sub-anesthetic dose of ketamine is known to elicit an increase in glucose metabolism in the SMA in TRD patients^73^, which is associated with ketamine’s antidepressant effect^74^. Similarly, the current results suggest that repeated doses of ketamine normalize the dysfunction of the SMA in remitters towards patterns observed in controls, whereas non-remitters who do not have this disruption at baseline show an opposite trend (diverge from controls) with ketamine infusion. Observations of lower BOLD activity in remitters in the SMA at baseline also suggest that a compensation of pretreatment function occurs with successful treatment, which is potentially facilitated by more lasting modulation of glutamatergic neurotransmission^74,75^. These findings thus suggest that BOLD activation in the SMA area during response inhibition could be a potential biomarker of ketamine treatment.

Previous BOLD fMRI studies of IV ketamine in depression have failed to report relationships between changes in functional imaging measures and clinical improvement when both are examined continuously. However, when binarizing patients into responder and non-responder groups, a recent investigation found increased global connectivity in the prefrontal cortex, caudate and insula in treatment responders only, suggesting changes in prefrontal and striatal circuitry may be relevant to successful outcomes^10^. In contrast, here we observed significant and robust negative associations between change in HDRS scores and change in BOLD activity in brain regions associated with remission during response inhibition after serial ketamine treatment. Furthermore, baseline and change in BOLD activity after acute treatment in the bilateral SMA correlated with end of treatment HDRS scores. These findings suggest that the SMA is a key node in the response-inhibitory network for TRD cases and future studies could elucidate how these functional changes in the SMA relate to the molecular mechanism underlying ketamine’s rapid antidepressant effects.

Several limitations should be acknowledged for the current investigation. Firstly, the limited number of HC (*n* = 32) led to reduced statistical power for cross-sectional comparisons at the whole-brain level, though at the regional level patients exhibited higher BOLD activity during response inhibition as compared to HC. Additional study limitations include that HCs were not measured twice. Nonetheless, it is important to note that the focus of this investigation was on change in neural response over time where subjects serve as their own controls. Previous studies have shown the NoGo activity^22,76^ in the anterior cingulate to be associated with clinical response. However, in the current study we do not observe this behavior since we evaluate the NoGo>Go contrast that provides higher statistical power and includes sensitivity to the motor response. Further, participants were allowed to continue concurrent stable anti-depressant medication, which may have impacted findings. Finally, this mechanistic study included open-label administration of ketamine without a placebo control. It is thus possible that placebo effects may have influenced results. However, since the objective here was to investigate perturbation of neural networks associated with ketamine rather than to address clinical efficacy, this limitation may of be of less impact for biological findings.

Taken together, our results demonstrate that repeated low-dose ketamine therapy leads to neurofunctional plasticity in regions essential for executive function, and inhibitory control in particular. Further, functional plasticity in SMA network after ketamine treatment are shown to relate to improvements in depressive symptoms, suggesting modulation of this network plays an important role in therapeutic response. In addition, results suggest that early changes in the inhibitory control network responsible for motor control are predictive of overall treatment outcome, suggesting these activation patterns may serve as a potential biomarker of treatment response. Future studies may expand upon the current findings, including addressing how treatment-related changes in the inhibitory control network vary in relation to longer term clinical outcomes, maintenance of therapeutic response and relapse.

## Supplementary information

Supplementary Table 1

Supplementary Figure 1
