# Supplementary material for: Modulation of inhibitory control networks relate to clinical response following ketamine therapy in major depression
Source: Transl Psychiatry. 2020 Jul 30;10:260. doi: 10.1038/s41398-020-00947-7 (PMC7393172; doi:10.1038/s41398-020-00947-7)
Supplement: Supplementary file 1 — Supplementary Table 1 [file 41398_2020_947_MOESM1_ESM.docx]

Supplementary Table 1: Current Medication
